# Next-Generation Sequencing in Tumor Diagnosis and Treatment

**DOI:** 10.3390/diagnostics10110962

**Published:** 2020-11-17

**Authors:** Dario de Biase, Matteo Fassan, Umberto Malapelle

**Affiliations:** 1 Department of Pharmacy and Biotechnology (FaBiT), Molecular Pathology Laboratory, University of Bologna, 40138 Bologna, Italy; 2 Department of Medicine (DIMED), Surgical Pathology Unit, University of Padua, 35121 Padua, Italy; 3 Department of Public Health, University of Naples Federico II, 80131 Naples, Italy

Next-Generation Sequencing (NGS) allows for the sequencing of multiple genes at a very high depth of coverage. The principle of targeted therapy consists of the identification of well-defined molecules that can be used as targets for drugs treatment, or as molecular markers that play a key role in tumor progression and/or survival. Considering the continuous discovery of new molecules as putative targets or that are responsible for treatment-resistance mechanisms, single-gene diagnostics is becoming less effective. Nowadays, precision medicine requires multigene characterization. The introduction of NGS to molecular diagnostics has allowed us to combine multigene analysis with high analytical sensitivity.

Many multi-gene panels have become commercially available in recent years. However, these panels include a high number of targets or are usually designed for specific tumors or genes. Developing custom/laboratory-developed multi-gene panels allows for the selection of targets, according to the needs of the medical community, serviced by the molecular laboratory. These panels allow for the optimization of the number of specimens that can be analyzed in a single run to reduce costs [1].

Another important aspect that is worth considering when NGS somatic analysis is performed using large panels, whole-exome or whole-genome sequencing, is that crucial germline findings for individuals and their families may also be revealed, in terms of preventing heritable cancer [2]. For this reason, clinicians should give their patients clear information about the possibility and benefit of knowing about a germline mutation for themselves and their relatives and written informed consent should be obtained. In the case of patients with germline mutations in actionable genes predisposed to cancer, an appointment with a geneticist or a genetics counselor should be strongly encouraged.

A solid-tumor NGS-based gene panel may allow for the analysis of mutational patterns, potential common features and may highlight the potential clonal relation of simultaneously occurring malignancies. An NGS approach identified pathogenic variants of 41 genes in carcinomas associated with Hashimoto’s thyroiditis, and several genes harbored multiple variants. Identical gene variants represented in both synchronous tumors of the same thyroid gland were found only in a small fraction of cases [3]. Common gene variants were found in *BRAF* (1 case) and *JAK3* (1 case); however, each tumor pair featured several other additional dominant variants, which were unrelated [3]. This massive parallel analysis suggests the presence of characteristic genetic patterns for different thyroid carcinoma etiologies.

Massive parallel sequencing also allows us to genetically characterize the profiles of tumor entities that are largely unknown, such as those of rare neoplasms. Atypical cellular angiofibroma (ACA) and cellular angiofibroma with sarcomatous transformation (CAS) are rare entities of cellular angiofibroma that show atypical morphology features [4]. An NGS approach to these neoplasms allowed us to identify some molecular alterations that may occur in these tumors, other than the previously known deletion of the *RB1* gene. *TP53* mutations were found in two out of five cases with CAS morphology, in agreement with the sarcomatous transformation. This p53 deficiency may be involved in the tumorigenesis of these entities. Moreover, a plethora of unclear gene variants were identified, which deserve further investigation [4].

Studies of whole-genome and -exome sequencing provide a huge reservoir of data that can be deeply analyzed not only for the identification of single nucleotide variants (SNVs), but also for the analysis of coding (mRNA) and non-coding RNA (miRNA, lncRNA), in specific subsets of tumors.

Using the public RNA-Seq dataset from the National Center for Biotechnology Information’s (NCBI) Gene Expression Omnibus (GEO) repository, those genes that can be used to differentiate specific Gleason groups in prostate cancers have been identified [5]. This model identified six gene transcripts that are differentially expressed among the different Gleason scores. This machine-learning approach, allowing us to identify differentially expressed prostate cancer stage-specific transcripts, will not only provide more insight into predicted prognostic outcomes and the development of effective therapeutic strategies against prostate cancer progression, but also in other cancers, if properly validated.

Bioinformatic analysis may also be applied to the pathways of genes with different expressions related to tumor progression, and to identify short RNA (e.g., miRNA) and long non-coding RNA (lncRNAs) interactions with mRNA. An RNA sequencing analysis performed on renal cancer cell lines identified 652 protein-coding genes and 92 lncRNA genes with significantly different gene expressions between renal carcinoma cell lines derived from primary and metastatic sites [6]. Further analyses, performed using two bioinformatics tools, identified more than 100 statistically significant biological pathways. Other than miRNAs, the study identified that two lncRNA and three protein-coding genes were significantly associated with increased or decreased disease-free survival in patients with renal cell carcinoma [6].

The introduction of NGS diagnostic approaches into clinical practice is allowing us both to introduce molecularly driven treatment choices and to perform an integrated morphological and molecular characterization of the biospecimen. However, it should be taken into account that the huge amount of data obtained from an NGS analysis need to be properly managed to determine the information that is correct and useful. The European Society for Medical Oncology (ESMO) has recently outlined their guidelines for the use of Next-Generation Sequencing (NGS) for patients with metastatic cancers [7]. The ESMO guidelines highlight the evidence that NGS panel results may lead to the recommendation of off-label drugs and, for this reason, it is crucial to regulate NGS procedures on a national level. The guidelines recommend using tumor multigene NGS in non-squamous NSCLCs, prostate cancers, and cholangiocarcinomas [7]. However, large panels of genes can be used for other tumors if this method is not associated with extra costs (e.g., in colorectal cancers), or in clinical centers to accelerate cancer research and drug development through clinical trials. Moreover, ESMO acknowledges that a doctor, together with the patient, may decide to require an NGS panel of genes, without extra costs for the healthcare system, if the patient is informed about the putative benefits of this analysis [7].
